# Ozone protects against ischemia-reperfusion injury: molecular mechanisms

**DOI:** 10.3389/fphar.2026.1771985

**Published:** 2026-04-29

**Authors:** Guixu Liu, Xueyan Gong, Zhuang Liu, Shoushi Wang

**Affiliations:** 1 School of Anesthesiology, Shandong Second Medical University, Weifang, China; 2 Qingdao Center Hospital of Rehabilitation University, Operating Room, Qingdao, China; 3 Department of Anesthesiology, Qingdao Center Hospital of Rehabilitation University, Qingdao, China

**Keywords:** ozone, ischemia-reperfusion injury, ozone preconditioning, medical ozone treatment, anti-inflammatory, oxidative stress, organ protection

## Abstract

Ischemia-reperfusion (I/R) injury is a significant pathological phenomenon that affects the prognosis of various diseases in clinical settings, causing negative outcomes in multiple clinical conditions. Although early reperfusion after ischemia is crucial, it triggers excessive oxidative stress, inflammatory storm, ion imbalances, and various forms of programmed cell death, resulting in extensive tissue necrosis and severe functional damage. As a powerful oxidant, ozone exhibits paradoxical therapeutic effects when administered at controlled low doses. Low-dose ozone pretreatment targets multiple pathways to counteract major vicious cycles in ischemia-reperfusion injury. The protective effects of ozone primarily enhance cellular antioxidant systems (e.g., by upregulating SOD, HO-1, and GSH expression) and anti-inflammatory responses through activating the Nrf2 pathway. Concurrently, ozone reduces inflammation by inhibiting the TLR4/NF-κB signaling axis and regulates immune cell responses. Ozone inhibits key cell death pathways (including apoptosis, ferroptosis, pyroptosis, etc.) by altering caspase activity, Bcl-2/Bax ratios, and NLRP3 inflammasome activation, while enhancing mitochondrial protection. Moreover, the protective mechanisms of ozone against ischemia-reperfusion injury via pathways such as MAPK may represent another significant research direction. This review summarizes the protective effects of ozone against I/R injury across various organs and provides insights into the underlying mechanisms. Furthermore, we explore the potential clinical applications of ozone in the treatment of I/R injury. The studies outlined in this review provide theoretical support for the use of ozone in the treatment of I/R injury and offer new perspectives for clinical applications.

## Introduction

Ischemia is defined as the “suffocation” of tissues or organs due to insufficient or interrupted blood perfusion. Pathological processes such as myocardial infarction (MI), cerebral infarction (CI), organ transplantation, and limb injuries can induce ischemia. While timely and effective reperfusion is a crucial therapeutic strategy for ischemia, it may cause additional damage to ischemic tissues and organs, a phenomenon known as ischemia-reperfusion (I/R) injury (Wu et al., 2018). Parks et al. suggest that reperfusion injury may be more serious than simple ischemic injury, capable of causing acute organ damage, organ failure, and life-threatening conditions (Parks and Granger, 1986). Several factors contribute to I/R injury, including sustained production of free radicals and reactive oxygen species (ROS), excessive inflammation, disruption of intracellular ion homeostasis, and cellular apoptosis and necrosis (Parks and Granger, 1986; Di Filippo et al., 2010a; Wu et al., 2018; Eskla et al., 2022; Xu et al., 2025). I/R-induced damage can be counteracted by ischemic preconditioning, hyperbaric oxygen therapy, hypothermia, and the use of anti-inflammatory and antioxidant agents such as melatonin (Peralta et al., 2002; Chen et al., 2008c; Gao et al., 2008; Altinel et al., 2011; Francis and Baynosa, 2017; Wang et al., 2021; Eskla et al., 2022).

In 1839, Christian Friedrich Schönbein discovered ozone, which he described as a pungent gas with an “electric taste” (Bocci et al., 2009). Ozone (O_3_) exists naturally in the atmosphere, particularly in the stratosphere (Mustafa, 1990). The weak bond of the third oxygen atom is responsible for ozone’s strong oxidizing properties, enabling its use in sterilization and disinfection (Mustafa, 1990; Bocci et al., 2009). Initially, ozone was viewed solely as a pollutant and harmful gas, with numerous studies demonstrating that prolonged or acute exposure to ozone can cause damage to the respiratory system and extrapulmonary organs (Mustafa, 1990; Tager et al., 2005; Taylor-Clark et al., 2009; Atkinson et al., 2016). However, Wolff et al. were able to show that ozone can function as a “natural medicine” and a potential therapeutic agent for numerous diseases (Zanardi et al., 2016; Wolff, 1979). Ozone is now widely used in clinical applications in the form of gas, ozonated water, ozonated oil, ozone autohemotherapy, and other formulations, primarily generated by corona discharge and electrolysis methods (Kogelschatz et al., 1988; Bocci et al., 2009). Furthermore, safe administration of ozone at precise doses can induce mild oxidative stress and activate the antioxidant machinery, thereby improving redox balance and inflammatory responses, and ultimately increasing the resistance of tissues and organs to I/R injury (Zamora et al., 2005; Chen et al., 2008a; Meng et al., 2017; Süzen Çaypınar et al., 2022). Currently, the protective effect of ozone against ischemia-reperfusion injury primarily operates through pretreatment strategies similar to ischemic preconditioning and hyperbaric oxygen therapy. Particularly, it exhibits many similar mechanisms to hyperbaric oxygen pretreatment, though there are also differences (Altinel et al., 2011; Xing et al., 2015; Meng et al., 2017; Wang R. et al., 2022; Zhang et al., 2022).

In this review, we have summarized the therapeutic effects of ozone against tissue and organ I/R injury, along with the underlying mechanisms. Furthermore, we have explored the potential clinical applications and challenges of ozone in the treatment of I/R injury. By synthesizing the latest findings in ozone loading research, we provide a reference for future clinical use and drug development.

## Mechanisms of I/R injury and the biological effects of ozone

### Mechanisms of I/R injury

I/R injury involves a multi-step, multi-pathway cascade of amplification, with common and organ-specific characteristics. First, tissue ischemia impairs oxygen and nutrient supply, driving cells to switch to anaerobic glycolysis, resulting in lactate accumulation, mitochondrial ATP depletion, and reduced antioxidant production (Wu et al., 2018; Baidya et al., 2020). High levels of lactic acid lead to significant retention of hydrogen ions (H^+^), which, in turn, triggers metabolic acidosis (Wu et al., 2023). Furthermore, ATP deficiency renders the ATP-dependent sodium-potassium (Na^+^-K^+^) and calcium (Ca^2+^) pumps, and the K-ATP channel dysfunctional, leading to intracellular accumulation of H^+^, Na^+^ and Ca^2+^, and extracellular K^+^ overload (Nauta et al., 1991; Murphy and Steenbergen, 2008). These hyperosmolar conditions induce cell swelling, acidosis, enzyme inactivation, chromatin condensation, and ribosomal dissociation, eventually leading to cell death (Wu et al., 2018). ATP deficiency also disrupts mitochondrial K-ATP channels, leading to mitochondrial damage (Roseborough et al., 2006; Wang et al., 2020). Furthermore, ATP degradation under these low-energy conditions leads to the accumulation of hypoxanthine, a breakdown product of ATP. Concurrently, the Ca2^+^ influx converts xanthine dehydrogenase to xanthine oxidase, thereby setting the stage for a free radical surge during reperfusion (Hoffman et al., 2004; Neuhof, 2014).

The sudden restoration of blood and oxygen supply to the tissues and organs following reperfusion, coupled with increased xanthine oxidase activity and leakage within the mitochondrial electron transport chain, triggers the production of ROS such as superoxide anion (O_2_^−^•), hydrogen peroxide (H_2_O_2_), and hydroxyl radical (•OH) (Murphy, 2009; Kura and Slezak, 2024). Simultaneously, ischemia leads to a deficiency in the cofactor tetrahydrobiopterin (BH_4_), preventing nitric oxide synthase (NOS) from producing nitric oxide (NO) (i.e., NOS decoupling). Instead, NOS generates O_2_•^-^, further exacerbating oxidative stress (Landmesser et al., 2003). The massive production of ROS far exceeds the scavenging capacity of superoxide dismutase (SOD), glutathione (GSH), and other antioxidant intermediates, resulting in oxidative stress that promotes endothelial dysfunction, DNA damage, and local inflammation (Tabuchi et al., 2010; Onal et al., 2015; Peng et al., 2025). The inflammatory cascade and oxidative stress may subsequently induce a cytokine storm, leading to cellular structural damage and ultimately cell death (Wu et al., 2018; Dugbartey, 2024). The intracellular Na^+^ accumulation during ischemia, and the massive pH gradient created between the intracellular and extracellular spaces after reperfusion, activate the reverse transport mode of the Na^+^-Ca^2+^ exchanger (Neuhof, 2014; Nakamura et al., 1999). This drives another wave of Ca^2+^ influx, which activates Ca^2+^-dependent proteases, phospholipase A_2_, and other enzymes, resulting in the degradation of cell membranes, cytoskeletal proteins, and DNA (Neuhof, 2014; Kura and Slezak, 2024). The combined effects of Ca^2+^ overload and oxidative stress lead to sustained opening of the mitochondrial permeability transition pore (mPTP), leading to membrane depolarization that stalls ATP synthesis, intensifies ROS production, and triggers the release of pro-apoptotic factors like cytochrome c (Cyt c) (Nauta et al., 1991; Ali et al., 2020; Nakamura et al., 1999). Oxidative damage to the sarcoplasmic reticulum and mitochondrial membranes causes leakage of the stored Ca^2+^. However, energy depletion prevents Ca^2+^ pumps from effluxing cytoplasmic Ca^2+^, leading to further Ca^2+^ overload and forming a vicious cycle (Nakamura et al., 1999). Mitochondrial dysfunction, endoplasmic reticulum stress, and disruption of cellular structure and function collectively drive programmed cell death, including apoptosis, necrosis, autophagy, and ferroptosis, ultimately amplifying tissue injury. This process is regulated by the caspase, PI3K/Akt, TLR/NF-κB, NLRP3 inflammasome, and JAK-STAT signaling pathways (Li et al., 2000; Murphy, 2009; Aydos et al., 2014; Yu et al., 2021; Li G. et al., 2022).

The cellular damage and stress responses induced by I/R injury trigger the release of numerous damage-associated molecular patterns (DAMPs), including high mobility group box protein B1 (HMGB1), heat shock proteins (HSPs), uric acid, ATP, etc. (Wang et al., 2024). The DAMPs bind to specific pattern recognition receptors (PRRs) like Toll-like receptor 4 (TLR4) on immune cell surfaces, and activate the pro-inflammatory nuclear factor κB (NF-κB) signaling pathway, leading to the synthesis of inflammasome proteins (e.g., NLRP3) and interleukin (IL) precursors (e.g., pro-IL-1β) (Gong et al., 2018; Wang et al., 2024). Simultaneously, reperfusion-induced ROS and Ca^2+^ overload also trigger the production and activation of NLRP3 and other inflammasomes, which in turn activate inflammatory cytokines such as tumor necrosis factor (TNF)-α and IL-1β through the caspase pathway (Neuhof, 2014; Gong et al., 2018; Wang L. et al., 2022). The immune cells recruited by these cytokines produce additional inflammatory mediators, resulting in a positive feedback loop that exacerbates inflammation (Nakamitsu et al., 2001). Free radicals and Ca^2+^ overload also induce the expression of adhesion molecules on endothelial cells, thus facilitating the migration and infiltration of neutrophils into damaged tissues (Di Filippo et al., 2010b; Gong et al., 2018). This triggers the release of additional inflammatory mediators and ROS, creating a vicious cycle that ultimately culminates in an “inflammatory storm.” Concurrently, complement system activation, oxidative stress stimulation, and reduced NO production further amplify the inflammatory response (Wang L. et al., 2022). All of the above mechanisms activate endothelial cells and promote infiltration of leukocytes, which release proteases (such as elastase) that directly damage parenchymal cells and microvascular structures (Neuhof, 2014). The ensuing swelling and detachment of endothelial cells promote the secretion of TNF-α and IL-1β, along with microthrombus formation, leading to microcirculatory impairment and the “no-reflow phenomenon”, which exacerbates circulatory dysfunction and inflammatory responses, resulting in a vicious cycle (Kloka et al., 2023). Additionally, studies show that the human immune system and microbiota may also play a significant role in I/R injury.

**FIGURE 1:**
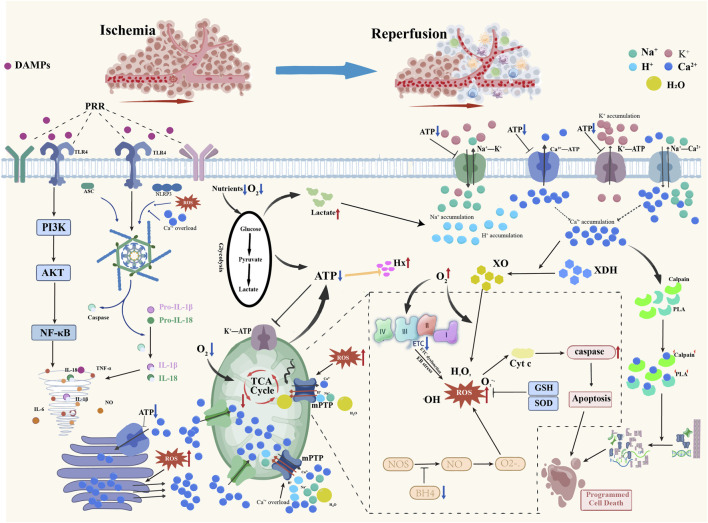
Schematic diagram of the mechanisms underlying ischemia-reperfusion injury. Ischaemia leads to elevated lactate levels and reduced ATP, which inhibits ion pump function, resulting in intracellular H^+^, Na^+^, and Ca^2+^ overload, extracellular K^+^ overload, and mitochondrial damage, while simultaneously causing the accumulation of hypoxanthine and xanthine oxidase. Sudden reperfusion causes leakage in the mitochondrial electron transport chain, generating large amounts of ROS. Concurrently, NOS uncoupling—prevented by energy depletion during ischemia—leads to the production of substantial ROS, resulting in oxidative stress. Oxidative stress and intracellular ionic disturbances further activate Na^+^-Ca^2+^ reverse transport and damage to mitochondria and the endoplasmic reticulum, leading to further Ca^2+^ release and creating a vicious cycle. Concurrently, oxidative stress and Ca^2+^ overload trigger inflammatory cell infiltration, the release of inflammatory mediators, and the activation of inflammasomes such as NLRP3, resulting in severe inflammatory responses. Additionally, DAMPs activate PRRs, further exacerbating the inflammatory response and creating a vicious cycle that leads to an inflammatory storm. These ischemia- and reperfusion-induced ionic disturbances, inflammatory storm, and cellular damage collectively contribute to the cascade amplification effect of ischemia-reperfusion injury. Red arrows indicate upregulation or activation, while blue arrows indicate inhibition.

In summary, I/R injury constitutes a vicious cycle initiated by an energy crisis and driven by oxidative stress, calcium overload, inflammatory responses, and multiple cell death pathways (Figure 1). Although the mechanisms of I/R injury vary across different tissues and organs, redox imbalance, inflammatory storm, and ion imbalance remain key contributing factors. The clinical interventions for I/R injury, such as ischemic pre-conditioning and hypothermia, have not achieved satisfactory or effective outcomes. Therefore, it is crucial to understand its underlying mechanisms and implement targeted interventions accordingly.

### Biological effects of ozone

**FIGURE 2:**
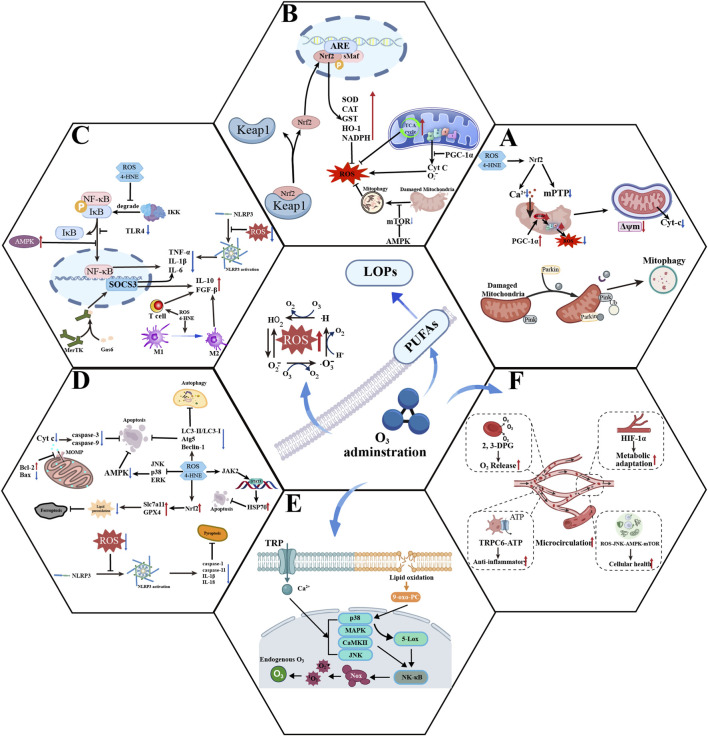
Schematic diagram of the biological effects of ozone. The central circle, representing an ozone molecule passing through the cell membrane, illustrates the generation of second messengers in the extracellular space following ozone administration. The two lower circles, indicated by arrows and not passing through the cell membrane, represent the biological effects of ozone acting directly on cells and blood. **(A)** Protective effects of ozone on mitochondria. Ozone clears damaged mitochondria via the PINK1/Parkin pathway, while simultaneously alleviating mitochondrial damage caused by Ca^2+^ overload and excessive mPTP opening. **(B)** Low-dose ozone primarily enhances the body’s antioxidant stress resistance and alleviates oxidative stress by activating the Nrf2 pathway, while also mitigating mitochondrial damage. **(C)** Ozone can suppress inflammatory responses caused by NF-κB activation and stimulate SOCS3 to further reduce inflammation. Ozone can also alleviate inflammatory responses by regulating immune cells. **(D)** Ozone reduces programmed cell death in organ cells and minimizes cellular damage by activating the Nrf2/HO-1 pathway, the Nrf2/Slc7a11/GPX4 pathway, and the AMPK/mTOR pathway, while inhibiting the MAPK pathway, JNK, p38, ERK, and others. It also regulates the expression of apoptosis-related proteins such as caspase-3, Bcl, and Bax. **(E)** Ozone can directly activate cationic TRP channels on the cell membrane and oxidize the cell membrane to produce bioactive lipid oxidation products, thereby activating the p38 MAPK pathway, JNK, and others, and generating endogenous reactive oxygen species to exert beneficial biohemical effects. **(F)** Ozone can improve tissue oxygenation, increase oxygen release by red blood cells, and improve microcirculation. Red arrows indicate upregulation or activation, while blue arrows indicate inhibition.

The theory of hormesis stipulates that low doses of harmful substances within controlled ranges may be beneficial to the human body (Bocci et al., 2011; Sagai and Bocci, 2011). As reported by Bocci in the same paper, the term hormesis was first described by L. Re in 2008 (Re et al., 2008). Ozone can be safely administered through local injection, intramuscular injection, intestinal insufflation, etc., while avoiding contact with the respiratory system and eyes (Bocci et al., 2009). The safe dose range in animal models is 10–80 μg/mL, demonstrating positive effects without toxicity. For humans, the safe dose of ozone ranges from 15 to 50 μg/mL (Taşçi Bozbaş et al., 2018; Setyo Budi et al., 2022; Li et al., 2024). When administered at low/therapeutic doses, ozone transiently reacts with bodily fluids and generates H_2_O_2_ and lipid oxidation products such as 4-hydroxy-2-nonenal (4-HNE) and malondialdehyde (MDA) at concentrations within the tolerable oxidative threshold (Bocci et al., 2009; 2011). These ROS molecules act as second messengers for multiple stress-responsive signaling pathways. Consequently, controlled oxidative stress activates the antioxidant system, reduces inflammatory responses, alleviates tissue hypoxia, and strengthens the immune system, resulting in enhanced resistance to I/R injury (Ajamieh et al., 2004; Chen et al., 2008a; Ahmed et al., 2012) (Figure 2).

#### Improvement in redox status

Low-dose ozone (typically 10–40 μg/mL) activates redox signaling networks by inducing mild oxidative stress, thereby producing a wide range of beneficial effects. Ozone-induced oxidative stress activates nuclear factor E2-related factor 2 (Nrf2), a transcription factor that regulates antioxidant and anti-inflammatory responses (Meng et al., 2017). Studies have confirmed that Nrf2 activation serves as the central mechanism underlying ozone’s multifaceted protective effects (Re et al., 2014; Galiè et al., 2018; Wang Z. et al., 2018; Galiè et al., 2019; Cisterna et al., 2020; Re, 2022; Ding et al., 2023; Zhu et al., 2024). Under normal conditions, Nrf2 is sequestered in the cytoplasm by its specific inhibitor Keap1, which promotes its degradation (Itoh et al., 1999; Galiè et al., 2018). However, after therapeutic-dose ozone acts on the body, the second messengers produced by the resulting oxidative stress promote the dissociation of Nrf2 from Keap1 and its translocation to the cell nucleus (Furukawa and Xiong, 2005; Pecorelli et al., 2013; Re et al., 2014; Galiè et al., 2018). This leads to the transactivation of genes driven by the antioxidant response element (ARE), thereby increasing the expression of genes whose promoters contain AREs (Furukawa and Xiong, 2005; Re et al., 2014; Scassellati et al., 2017; Galiè et al., 2018; Re, 2022). This process promotes the synthesis of a broad spectrum of cytoprotective and antioxidant enzymes (Pecorelli et al., 2013; Re et al., 2014; Galiè et al., 2018). Thus, Nrf2 activation upregulates genes encoding key antioxidant enzymes such as SOD, catalase (CAT), glutathione peroxidase (GPX), glutathione S-transferase (GST), heme oxygenase-1 (HO-1), reduced nicotinamide adenine dinucleotide phosphate (NADPH), and NADPH quinone oxidoreductase 1 (Galiè et al., 2019; Re, 2022). Ozone exposure also promotes the biosynthesis and regeneration of reduced GSH, bilirubin, and quinone compounds, thereby significantly enhancing cellular antioxidant capacity (Chen et al., 2008c; Koca et al., 2010; Cai et al., 2020). Furthermore, ozone can enhance cellular metabolism and oxidative stress tolerance by optimizing mitochondrial function through various mechanisms (Cai et al., 2020). Ozone exposure stimulates the tricarboxylic acid cycle by enhancing pyruvate oxidative decarboxylation, reducing NADH levels, and oxidizing cytochrome c, thereby decreasing ROS production and improving blood circulation (Ajamieh et al., 2005; Meng et al., 2017; Viebahn-Haensler and León Fernández, 2024). In addition, ozone reduces O_2_^−^• leakage in the electron transport chain (ETC) and promotes mitochondrial biogenesis through activation of the peroxisome proliferator-activated receptor gamma coactivator-1α (PGC-1α) signaling pathway, and inhibits the release of mitochondrial Cyt-c into the cytoplasm, all of which contribute to reduced ROS generation (Viebahn-Haensler and León Fernández, 2024). In the context of I/R injury, some studies suggest that ozone may help reduce post-reperfusion oxidative stress, possibly by decreasing the accumulation of hypoxanthine and xanthine and by inhibiting the xanthine/xanthine oxidase pathway. Furthermore, ozone plays a crucial role in maintaining cellular redox homeostasis by inducing endothelial NOS (eNOS) expression and stimulating NO synthesis (Chen et al., 2008c; Di Filippo et al., 2010b). Ozone-mediated activation of the AMPK pathway further stimulates the pro-antioxidant Nrf2/HO-1 signaling pathway. Simultaneous inhibition of downstream mechanistic target of rapamycin (mTOR) activity relieves the suppression of autophagy, thus enabling timely clearance of damaged mitochondria and proteins (Lee et al., 2003; Xiao et al., 2017; Wang Z. et al., 2018; Xu et al., 2021). This process further reduces ROS production and improves the redox state. In summary, ROS production and the disruption of cellular redox balance are the key triggers of I/R injury, and low-dose ozone can mitigate I/R-induced oxidative stress by stabilizing redox states.

#### Mitigation of inflammatory responses

The anti-inflammatory effects of low-dose ozone result from multi-pathway synergy. Rather than directly neutralizing inflammatory mediators, ozone induces moderate oxidative stress to activate a series of endogenous antioxidant defenses, modulates immune cell function, and improves tissue oxygenation and microcirculation, thereby achieving anti-inflammatory outcomes.

In the resting state, NF-κB is sequestered in the cytoplasm by the inhibitory protein IκB. The IκB kinase (IKK) complex is activated in response to a requisite stimulus and releases NF-κB for nuclear translocation by phosphorylating and degrading IκB (Hinz and Scheidereit, 2014). The secondary messengers generated by ozone, mainly H_2_O_2_ and 4-HNE, block the pro-inflammatory NF-κB pathway by inhibiting IKK (Li et al., 2000). Furthermore, ozone inhibits nuclear translocation of NF-κB by inactivating the TLR4 signaling pathway, thereby significantly decreasing the production of key pro-inflammatory cytokines such as TNF-α, IL-1β, and IL-6. The inhibitory action of ozone on the TLR4/NF-κB pathway protects against I/R injury (Xing et al., 2015; Wang Z. et al., 2018). Furthermore, ozone also increases the production of anti-inflammatory factors such as IL-10 and transforming growth factor (TGF)-β, which reshape the cytokine balance in inflamed tissues and mitigate the inflammatory response (Wang R. et al., 2022). The crosstalk between Nrf2 activation and NF-κB inhibition, and the suppressive effect of Nrf2 on ROS-induced NLRP3 activation, also yields anti-inflammatory effects (Wang Z. et al., 2018; Xu et al., 2025).

Ozone stimulates the production of Gas6, which binds to its receptor MerTK and activates cytokine-stimulated suppressor of cytokine signaling 3 (SOCS3), a key negative regulator of inflammatory responses (Ruan et al., 2024). Additionally, AMPK activation by ozone alleviates inflammation by suppressing NF-κB activity (Hinz and Scheidereit, 2014; Xing et al., 2015; Ruan et al., 2024). Low-dose ozone also exhibits unique immunomodulatory effects by bidirectionally regulating immune cells. For instance, ozone reprograms macrophages from the pro-inflammatory M1 phenotype to the anti-inflammatory M2 phenotype, enhancing their phagocytic function and the secretion of anti-inflammatory factors such as IL-10, thereby promoting inflammation resolution (Morales-Portillo et al., 2025). Simultaneously, ozone increases the abundance of regulatory T cells (Tregs) and activates them to release IL-10 and TGF-β. Finally, ozone also reduces the proportion of Th17 cells and inhibits the production of pro-inflammatory factors such as IL-6 and IL-17 (Chen et al., 2008a). This homeostasis between immunosuppressive and immunostimulatory T cells is crucial for controlling excessive immune responses. Ozone also activates the production of growth factors such as vascular endothelial growth factor (VEGF), platelet-derived growth factor (PDGF), and TGF-β, while inhibiting TNF-α production, suppressing Kupffer cell activation, and reducing neutrophil infiltration, thereby alleviating inflammation (Chen et al., 2008c).

Ozone-induced increase in heme oxygenase-1 (HO-1) and bilirubin exerts anti-inflammatory effects through direct and indirect mechanisms, respectively–HO-1 through its catalytic product carbon monoxide, and bilirubin via its antioxidant action (Lee et al., 2003; Sagai and Bocci, 2011).

Moreover, ozone improves local microcirculation and tissue oxygen supply by increasing the production of 2,3-diphosphoglycerate (2,3-DPG) within red blood cells (RBCs) and reducing the affinity of hemoglobin for oxygen, thereby promoting the release of oxygen from hemoglobin (Bocci et al., 2011). This alleviates hypoxia in inflamed areas and mitigates inflammation. Simultaneously, ozone-driven NO production not only exerts direct anti-inflammatory effects but also induces vasodilation and improves blood flow, thus promoting inflammation resolution (Chen et al., 2008c; Di Filippo et al., 2010b). Finally, ozone pretreatment also increases adenosine levels, which can block the activation of pro-inflammatory pathways (León Fernández et al., 2007). Taken together, ozone demonstrates potent anti-inflammatory effects through its multi-modal, multi-level, and multi-pathway synergistic mechanisms, exhibiting significant potential in managing I/R injury.

#### Inhibition of cell death and mitoprotection

Ozone reduces cell death and improves energy metabolism during I/R injury through anti-apoptotic and mito-protective effects that induce “oxidative preadaptation” (Chen et al., 2008c; Di Filippo et al., 2008). Medical ozone treatment increases levels of the anti-apoptotic protein Bcl-2, inhibits the pro-apoptotic protein Bax, and elevates the Bcl-2/Bax ratio, which prevents mitochondrial channel opening, suppresses Cyt c efflux, and impedes the caspase-3/9 cascade, thereby inhibiting apoptosis (Chen et al., 2008a; Wang L. et al., 2018; Cai et al., 2020; Zhang et al., 2022). Additionally, ozone can indirectly reduce caspase-3 activity by activating the Nrf2/HO-1 pathway (Sagai and Bocci, 2011; Wang Z. et al., 2018). Low-dose ozone also inhibits the synergistic phosphorylation of c-Jun N-terminal kinase (JNK), p38, and extracellular signal-regulated kinase (ERK), eventually inactivating the MAPK pathway and reducing cellular apoptosis (Lee et al., 2003; Wang L. et al., 2018; Carini et al., 2001). On the other hand, ozone has been shown to inhibit cardiomyocyte death in a dose-dependent manner by activating the JAK2/STAT3 pathway and upregulating HSP70 (Komarova et al., 2004; Yu et al., 2021). Pyroptosis is a pro-inflammatory form of programmed cell death that is morphologically similar to apoptosis and necrosis. Ozone can effectively protect renal cells during reperfusion by inhibiting the pyroptotic cascade involving the NLRP3 inflammasome, caspase-1, caspase-11, IL-1β, and IL-18 (Wang et al., 2019). Ferroptosis is a form of programmed cell death that is driven by iron accumulation and lipid peroxidation. Ozone has been shown to reduce lipid peroxidation products by upregulating solute carrier family 7 member 11 (Slc7a11) and GPX4 via Nrf2 (Ding et al., 2023; Zhu et al., 2024). Likewise, AMPK activation by ozone inhibits ferroptosis and apoptosis by suppressing mTOR phosphorylation (Xiao et al., 2017; Shi et al., 2019). Furthermore, ozone-mediated reduction in oxidative stress and inflammatory responses also lowers the rate of abnormal cell death, while improvements in microcirculation and metabolism further mitigate cellular damage. Concurrently, ozone activates the AMPK/mTOR pathway to upregulate LC3-II, Beclin-1, and ULK1 expression, thereby enhancing autophagic flux to clear damaged mitochondria and protein aggregates (Scassellati et al., 2020). In fact, ozone treatment can inhibit excessive autophagy and apoptosis following I/R by decreasing the LC3-II/LC3-I ratio and downregulating Atg5 and Beclin-1 (Xu et al., 2021).

Studies show that ozone promotes mitochondrial biosynthesis and improves mitochondrial quality by upregulating PGC-1α via the Nrf2/HO-1/NQO1 pathway (Lee et al., 2003; Cisterna et al., 2020). This subsequently increases the expression of NRF1 and the mitochondrial transcription factor ATFAM, resulting in increased mitochondrial DNA replication and the synthesis of, ETC proteins (Gureev et al., 2019; Li et al., 2025). Concurrently, enhanced Nrf2-mediated antioxidant capacity also reduces mitochondrial damage. Furthermore, ozone-induced mild oxidative stress may eliminate dysfunctional mitochondria by activating the PINK1/Parkin pathway (Foglieni et al., 2011). The role of the PINK1/Parkin pathway in regulating mitochondrial autophagy is well documented (Ivankovic et al., 2016). Ozone mitigates oxidative stress and calcium overload, diminishes cardiolipin peroxidation, and lowers mitochondrial membrane potential (ΔΨm) dissipation. This inhibits excessive mPTP opening, thereby preventing mitochondrial swelling and Cyt-c leakage (Naserzadeh et al., 2019). Furthermore, medical ozone treatment optimizes, ETC function by enhancing the activity of complexes I and IV and improving ATP synthesis (Zhang et al., 2025). This is particularly evident in ischemia or metabolic disease models, where increased oxygen consumption rate and ATP yield have been observed (Viebahn-Haensler and León Fernández, 2024). The mito-protective effect of ozone eventually reduces ROS production and protects cells from oxidative stress-induced damage. Altogether, medical ozone treatment can improve mitochondrial function while blocking abnormal cell death through various pathways, thereby enhancing the functional state of tissues and organs, thus offering a new therapeutic option for managing I/R injury (Ajamieh et al., 2005; Foglieni et al., 2011).

#### Other beneficial effects of ozone

In addition to exerting effects via second messengers, emerging evidence indicates that direct contact of medical ozone with cells can also induce beneficial outcomes (Onyango, 2023). These direct beneficial effects are attributed to the activation of transient receptor potential (TRP) channels, the generation of biologically active mediators through membrane lipid oxidation, and the induction and amplification of endogenous ozone formation within cells (Taylor-Clark and Undem, 2010; Zemski Berry and Murphy, 2016). Ozone directly activates cationic TRP channels at the cell membrane, such as TRPA1, TRPV1, and TRPC6, thereby triggering calcium influx (Taylor-Clark and Undem, 2010; Li et al., 2019; Li C. et al., 2022; Chen et al., 2020; Hu et al., 2021). This subsequently activates pro-survival signaling pathways, including CaMKII, MAPK, p38, and JNK (Taylor-Clark et al., 2009; Taylor-Clark and Undem, 2010; Wang W.-Y. et al., 2010). Furthermore, ozone directly reacts with unsaturated phospholipids and cholesterol in the cell membrane, generating bioactive lipid oxidation products (Serhan et al., 1984; Santrock et al., 1992; Pulfer and Murphy, 2004). For instance, ozone-oxidized membrane phospholipids (e.g., phosphatidylcholine) yield species like 1-palmitoyl-2-(9′-oxo-nonanoyl)-sn-glycero-3-phosphocholine (9-oxo-PC), which activates the p38 MAPK pathway and subsequently stimulates 5-lipoxygenase (5-Lox) to produce anti-inflammatory lipoxins (Santrock et al., 1992; Uhlson et al., 2002; Pulfer and Murphy, 2004; Wang W.-Y. et al., 2010; Zemski Berry and Murphy, 2016). Following cellular exposure to ozone, direct activation of membrane TRP channels or oxidation of membrane lipids can activate transcription factors such as NF-κB (Taylor-Clark and Undem, 2010; Togi et al., 2021). This leads to the upregulation of NADPH oxidase (Nox), resulting in the intracellular generation of superoxide anion (O_2_^−^•) and singlet oxygen (^1^O_2_), which further react to form endogenous ozone (Onyango, 2016; Wanjala et al., 2018).

Low-dose ozone increases 2,3-DPG production in RBCs by stimulating glycolysis, thereby enhancing the oxygen-release capacity of hemoglobin and improving tissue oxygenation (Bocci et al., 2011). Ozone can promote platelet release of ATP by directly activating channels, such as the transient receptor potential cation channel subfamily C member 6 (TRPC6) (Vemana et al., 2015; Paez Espinosa et al., 2019; Saqib et al., 2023). This ATP is subsequently metabolized into adenosine, which activates anti-inflammatory and vasodilatory pathways. Ozone also improves the rheological properties of blood by reducing plasma viscosity and RBC aggregation while enhancing RBC deformability. This optimizes microcirculation, ensuring efficient oxygen delivery to cells. Furthermore, increased NOS activity and endogenous NO production due to ozone promote vasodilation, which inhibits platelet aggregation and enhances microcirculatory perfusion and tissue oxygenation, thus overcoming the “no-reflow phenomenon.” (Chen et al., 2008c; Di Filippo et al., 2008; Cai et al., 2020). Low-dose ozone also stimulates capillary neogenesis and improves local blood circulation by upregulating VEGF, which accelerates wound healing and tissue regeneration. Furthermore, ozone-induced upregulation of hypoxia-inducible factor-1α (HIF-1α) during ischemia-hypoxia promotes glycolysis and angiogenesis, thus facilitating metabolic adaptation to alleviate an energy crisis (Wang R. et al., 2022). Therapeutic-dose ozone triggers a ROS-JNK-AMPK-mTOR signaling cascade that inhibits mTOR phosphorylation and blocks downstream anabolic signals. This shifts the cells from a proliferative mode to an energy-conservation mode and enhances cellular resilience. Additionally, ozone improves cellular metabolic health by optimizing mitochondrial function (Xiao et al., 2017; Zhao et al., 2018).

Based on current research, it is evident that prolonged, high-dose, or improper exposure to ozone can cause pulmonary and systemic damage (Enweasor et al., 2021). On the other hand, low-dose ozone can activate the endogenous antioxidant systems, modulate inflammatory responses, reduce cell death, protect mitochondrial function, maintain immune homeostasis, and improve circulation and metabolism (Peralta et al., 1999; Di Filippo et al., 2010b; Xing et al., 2015; Setyo Budi et al., 2022; Wang R. et al., 2022). These positive effects of ozone can effectively mitigate the pathophysiological changes associated with I/R injury. Furthermore, the molecular mechanisms of therapeutic-dose ozone consistently center on the Nrf2 pathway, which also exerts significant influence on cellular processes (Meng et al., 2017; Zhu et al., 2024).

## Ozone’s ischemic reperfusion protection for various organs

**FIGURE 3:**
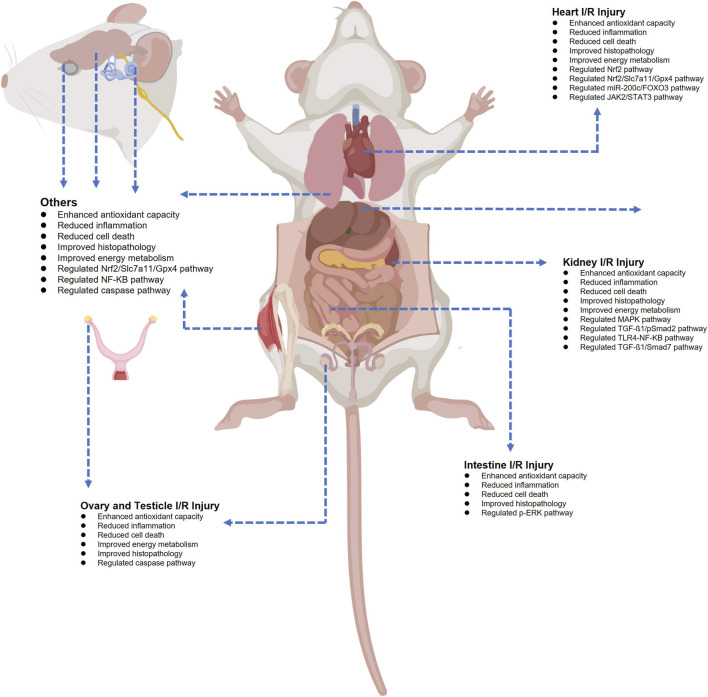
Ozone can protect many vital organs from ischemia-reperfusion injury. Ozone inhibits or alleviates ischemia-reperfusion injury by regulating various key signaling pathways and genes in multiple organs, including the heart, liver, kidneys, intestines, brain, testes, and uterus. The figure illustrates the mechanisms by which ozone alleviates ischemia-reperfusion injury, as confirmed by existing research.

Ozone has demonstrated a surprising array of therapeutic effects owing to its oxidizing properties. Furthermore, a substantial body of research indicates that medical ozone treatment can mitigate I/R injury in the heart, kidneys, liver, intestines, gonads, brain, lungs, skeletal muscle, skin, eyes, and ears. Despite the strong experimental evidence, numerous challenges remain in translating the therapeutic effects of ozone into clinical applications. Furthermore, inappropriate ozone administration may fail to deliver anticipated benefits or even induce additional injury. Table 1–3 summarizes the effects of rational ozone pretreatment on I/R injury across major organs, along with associated alterations in pathways and cytokine changes. Figure 3 summarizes the effects of ozone on I/R injury in major organs and the underlying mechanisms.

**TABLE 1:** Summary of studies investigating the effects of ozone on Heart I/R injury.

Animal/Cell	Model	Ozone concentration and dosage	Intervention measures (time/Method)	Sample collection time	Mechanism of action	Author/Article
SD rat	LAD I/R model	Concentration:20, 30, 40 μg/mL Dosage:1 mg/kg	Preconditioning (5 days before ischemia/p.r.)	2 h after reperfusion	Inhibite NLRP3 expression	Xu et al. (2025)
Wistar rat	Diabetes LAD I/R model	Dosage:1 mg/kg	Therapeutic intervention (5 min before reperfusion/i.p.)	5 min after reperfusion	Reduce myocardial cell apoptosis; enhance antioxidant capacity	Gülcan et al. (2024)
C57BL/6 mice, H9C2 cell	Mice: LAD I/R model; cell: H/R model	Concentration: Mices:25 μg/mL; Cells:20 μg/mL dosage: Mices:2 mL/day	Preconditioning (Mices: 5 days before ischemia/i.p.; Cells: 12 h before ischemia/Ozonated Culture Medium)	Mices:2 h after reperfusion cells: 3 h after reoxygenation	Activate Nrf2/Slc7a11/GPX4 pathway; inhibite ferroptosis; enhance antioxidant capacity	Ding et al. (2023)
Neonatal mouse cardiomyocyte	H/R model	Concentration:5, 10, and 20 μg/mL	Preconditioning (4 h before ischemia/O₂/O₃ mixed gas culture)	6 h after reoxygenation	Regulate miR-200c/FOXO3 pathway	Zhang et al. (2022)
Rabbit	Langendorff perfusion for isolated heart	Concentration:20, 40, 80 μg/mL Dosage:10 mL	Preconditioning (5 days before ischemia/i.p.)	Multiple timepoints:Before ischemia, 5 min after reperfusio, 60 min after reperfusion	Upregulate HIF-1α; anti-inflammatory; reduce mitochondrial dysfunction	Wang L. et al. (2022)
H9C2 cell	OGD/R model	Concentration:10, 20, 40, 60 μg/mL	Therapeutic intervention (Immediately following reperfusion/Ozonated Culture Medium)	Immediately after reoxygenation end	Inhibite autophagy	Xu et al. (2021)
SD rats; Neonatal rat cardiomyocyte	Rat: LAD I/R model; cell: H/R model	Concentration: Cells:10、20 μg/mL; dosage: Rats:50、100 μg/kg/day	Preconditioning (Rats:5 days before ischemia/i.p.; Cells:12 h before ischemia/Ozonated Culture Medium)	Rats:Immediately after reperfusion endCells:Immediately after reoxygenation end	Activate the JAK2/STAT3 pathway; upregulate HSP70 expression; inhibite apoptosis	Yu et al. (2021)
SD rat	LAD I/R model	Dosage:100 μg/kg/day	Preconditioning (5 days before ischemia/i.p.)	Immediately after reperfusion end	Activate the Nrf2 pathway; reduce mitochondrial damage and apoptosis; enhance antioxidant capacity	Meng et al. (2017)
Wistar rat	LAD I/R model	Concentration:low:15→25 μg/mL; high:30→50 μg/m Dosage:low:0.3→0.5 mg/kg/day; high:0.6→1.0 mg/kg/day	Preconditioning (2 weeks before ischemia/p.r.)	Immediately after reperfusion end	Improve histopathology; enhance antioxidant capacity	Ahmed et al. (2012)
SD rat	LAD I/R model	Dosage:100, 150, 300 μg/kg	Preconditioning (1 h before ischemia/i.p.)	2 h after reperfusion	Increase eNOS activity; Recruite endothelial progenitor cells	Di Filippo et al. (2010a)
SD rat	Arrhythmia model	Dosage:100, 150, 300 μg/kg	Preconditioning (31 s before ischemia/i.p.)	2 h after reperfusion	Stabilize ion channels and antiarrhythmic; Antioxidant	Di Filippo et al. (2010b)
SD rat	LAD I/R model	Dosage:100, 150, 300 μg/kg	Preconditioning (1 h before ischemia/i.p.)	2 h after reperfusion	Reduce nitrosative stress, inflammation, immune responses and apoptosis	Di Filippo et al. (2008)
Isolated rat heart	Langendorff model	Concentration:30 μg/mL Dosage:0.85 mL	Therapeutic intervention (5 min after reperfusion began/infusion)	Multiple timepoints:Before ischemia, every 10 min after reperfusion (40 min)	Enhance cardiac recovery after reperfusion(Enhance NO and regulated oxygen free radicals)	Merin et al. (2007)

LAD, left anterior descending; p.r.,per rectum; i.p.,intraperitoneal injection; H/R, Hypoxia/Reoxygenation; OGD/R,Deprivation/Reperfusion.

**TABLE 2:** Summary of studies investigating the effects of ozone on liver, kidney, and intestine I/R injury.

Organ	Animal/Cell	Model	Ozone concentration and dosage	Intervention measures (time/Method)	Sample collection time	Mechanism of action	Author/Article
Liver	Wistar rat	PHx model & whole liver I/R model	Concentration:60 μg/mL Dosage:1.2 mg/kg	Preconditioning (5 days before ischemia/i.p.)	24 h after reperfusion, 48 h after reperfusion	Enhance antioxidant capacity; anti-inflammatory; promote liver regeneration; regulate the NF-κB signaling pathway	Gultekin et al. (2013)
	Wistar rat	Right liver lobe I/R model	Concentration:50 μg/mL Dosage:1 mg/kg	Preconditioning (15 days before ischemia/p.r.)	Preconditioning end, Immediately after ischemia, 90 min after reperfusion	Enhance NO, antioxidant capacity; activate A₁ adenosine receptors; regulate the NF-κB signaling pathway and HSP-70 expression	León Fernández et al. (2008)
	Wistar rat	Right liver lobe I/R model	Concentration:50 μg/mL Dosage:1 mg/kg	Preconditioning (15 days before ischemia/p.r.)	Immediately after reperfusion, 90 min after reperfusion	Increase Mn-SOD activity; enhance antioxidant capacity; improve mitochondrial function	Ajamieh et al. (2005)
	Wistar rat	Right liver lobe I/R model	Concentration:50 μg/mL Dosage:1 mg/kg	Preconditioning (15 days before ischemia/p.r.)	Immediately after reperfusion, 90 min after reperfusion	Regulate NO production; enhance antioxidant capacity	Ajamieh et al. (2004)
	Wistar rat	Right liver lobe I/R model	Concentration:50 μg/mL Dosage:1 mg/kg	Preconditioning (15 days before ischemia/p.r)	Preconditioning end, Immediately after ischemia, 90 min after reperfusion	Enhance antioxidant capacity; reduce ATP degradation and uric acid production; improve histopathology	Ajamieh et al. (2003)
	Wistar rat	Right liver lobe I/R model	Concentration:50 μg/mL Dosage:1 mg/kg	Preconditioning (15 days before ischemia/p.r.)	Preconditioning end, Immediately after ischemia, 90 min after reperfusion	Enhance antioxidant capacity; inhibite XO; improve histopathology	Ajamieh et al. (2002)
	Wistar rat	Right liver lobe I/R model	Concentration:50 μg/mL Dosage:1 mg/kg	Preconditioning (10 days before ischemia/p.r.)	90 min after ischemia, 90 min after reperfusion	Enhance adenosine; inhibite XXO pathway; enhance antioxidant capacity	Peralta et al. (2000)
Kidney	Wistar rat	Right nephrectomy & left renal I/R model	Dosage: 0.5 mg/kg	Therapeutic intervention(Immediately administer after reperfusion/p.r.)	10 days after reperfusion, 12 weeks after reperfusion	Inhibite the TGF-β1/pSmad2 pathway; enhance antioxidant capacity; reduce inflammation and tubulointerstitial fibrosis	Jiang et al. (2020)
	SD rat; NRK-52E cell	Rats:Right nephrectomy & left renal I/R Model; cells:H/R model	Concentration: Cell: 20, 30, 40 μg/mL dosage: Rats: 1, 2 mg/kg	Therapeutic intervention (Immediately administer after reperfusion/p.r.)	Rats:10 days after reperfusionCells:Immediately after reoxygenation end	Inhibite the MAPK signaling pathway; enhance antioxidant capacity; inhibite apoptosis	Wang Z. et al. (2018)
	SD rat; NRK-52E cell	Rats:Right nephrectomy & left renal I/R model; Cells:H/R model	Dosage: 2 mg/kg	Therapeutic intervention (Immediately administer after reperfusion/i.p.)	24 h after reperfusion	Inhibite pyroptosis	Wang L. et al. (2018)
	Wistar rat	Right nephrectomy & left renal I/R model	Concentration:50 μg/mL Dosage: 1 mg/kg	Preconditioning (15 days before ischemia/p.r.)	24 h after reperfusion	Inhibite the TLR4-NF-κB pathway; anti-inflammatory	Xing et al. (2015)
	SD rat	Right nephrectomy & left renal I/R model	Concentration:50 μg/mL Dosage: 1 mg/kg	Preconditioning (15 days before ischemia/p.r.)	8 weeks after reperfusion	Inhibite the TGF-β1/Smad7 pathway and fibrosis-related proteins	Wang et al. (2014)
	SD rat	Bilateral renal I/R model	Concentration:60 μg/mL Dosage: 0.7 μg/kg	Therapeutic intervention (Immediately administer after reperfusion/p.r.)	6 h after reperfusion	Inhibite nitrosative stress; enhance antioxidant capacity	Oztosun et al. (2012)
	Wistar rat	Right nephrectomy & left renal I/R model	Concentration:50 μg/mL Dosage:1 mg/kg	Preconditioning (15 days before ischemia/p.r.)	24, 48, 72 h after reperfusion	Increase eNOS and iNOS expression; enhance antioxidant capacity; inhibite ET-1	Chen et al. (2008a)
	Wistar rat	Right nephrectomy & left renal I/R model	Concentration:50 μg/mL Dosage:1 mg/kg	Preconditioning (15 days before ischemia/p.r.)	24 h after reperfusion	Increase eNOS and iNOS expression; enhance antioxidant capacity	Chen et al. (2008b)
	Wistar rat	Right nephrectomy & left renal I/R model	Concentration:50 μg/mL Dosage:1 mg/kg	Preconditioning (15 days before ischemia/p.r.)	24 h after reperfusion	Anti-inflammatory; inhibite the caspase pathways; inhibite apoptosis	Chen et al. (2008c)
Intestine	SD rat	SMA and PV I/R model	Concentration: 50%/50% dosage: 0.7 mg/kg/day	Therapeutic intervention(Immediately administer after reperfusion/i.p. & p.r.)	48 h after reperfusion	Enhanced antioxidant capacity; enhanced cell proliferation; inhibited apoptosis; activated the p-ERK signaling pathway	Haj et al. (2014)
	SD rat	Acetate-induced acute distal colitis model	Concentration:60 μg/mL Dosage:0.7 mg/kg	Therapeutic intervention(Immediately administer after reperfusion/i.p.)	5 days after reperfusion	Anti-inflammatory; enhance antioxidant capacity	Altinel et al.(2011)
​	SD rat	SMA I/R model	Concentration:25 μg/mL Dosage: 0.5 mg/kg	Therapeutic intervention (45 min before reperfusion/i.p.)	1 h after reperfusion	Enhanced antioxidant capacity; improved histopathology	Isik et al.(2015)
	Wistar rat	SMA I/R model	Concentration:5% O₃ + 95% O₂ Dosage:0.7 mg/kg	Therapeutic intervention (20 min before reperfusion/i.p.)	1 h after reperfusion	No significant protective effect	Dere Günal et al. (2020)
	SD rat	SMA I/R model	Concentration:60 μg/mL Dosage: 0.7 mg/kg	Therapeutic intervention (30 min before reperfusion/i.p.)	1 h after reperfusion	Reduced endoplasmic reticulum stress; enhanced antioxidant capacity; improved histopathology	Demiral et al. (2024)
	Wistar rat	SMA I/R model	Dosage:1 mg/kg	Therapeutic intervention (Immediately administer after reperfusion/i.p.)	24 h after reperfusion	Enhanced antioxidant capacity; improved histopathology; anti-inflammatory	Erginel et al. (2023)
	Wistar rat	SMA I/R model	Concentration:60 μg/mL Dosage:1 mg/kg	Preconditioning (5 days before ischemia/i.p.)	2 h after reperfusion	Enhanced antioxidant capacity; improved histopathology	Onal et al. (2015)

PHx, Partial Hepatic Resection; i.p., intraperitoneal injection; p.r., *per rectum*; NO, nitric oxide; XO, xanthine oxidase; XXO, Xanthine/Xanthine Oxidase; SMA, superior mesenteric artery; PV, portal vein.

**TABLE 3:** Summary of studies investigating the effects of ozone on other organs I/R injury.

Organ	Animal/Cell	Model	Ozone Concentration and Dosage	Intervention Measures (Time/Method)	Sample collection time	Mechanism of Action	Author/Article
Ovary	Wistar rat	OT I/R model	Concentration:25 μg/mL Dosage:0.5 mg/kg	Preconditioning (10 min before reperfusion/i.p.)	2 h after reperfusion	Enhanced antioxidant capacity; enhanced NO; improved histopathology	Aslan et al. (2012)
	Wistar rat	OT I/R model	Concentration:95% O₂ & 5% O₃ Dosage:1 mg/kg	Therapeutic intervention (15 min before reperfusion/i.p.)	7.15 days after ischemia	Enhanced antioxidant capacity; improved histopathology	Süzen Çaypınar et al. (2022)
	SD rat	OT I/R model	Concentration:25 μg/mL Dosage:0.5 mg/kg	Preconditioning (45 min before reperfusion/i.p.)	2 h after reperfusion	Enhanced ATP and 2,3-DPG; improved glycolysis and peripheral tissue oxygenation	Sayar et al. (2016)
Testicles	Wistar rat	TT I/R model	Concentration:50 μg/mL Dosage:4 mg/kg	Preconditioning (1.5 h before reperfusion/i.p. or testicular injection)	4 h after reperfusion	Enhanced antioxidant capacity	Mete et al. (2017)
	Wistar rat	TT I/R model	Concentration:96% O₂ + 4% O₃ Dosage:1 mg/kg	Therapeutic intervention (Immediately administer after reperfusion/i.p.)	7 days after reperfusion	Enhanced antioxidant capacity; improved histopathology	Karlı et al. (2023)
	Wistar rat	TT I/R model	Dosage:1 mg/kg	Preconditioning (15 min before reperfusion/i.p.)	4 h after reperfusion	Inhibited TNFR, reduced eNOS and NO; inhibited the caspase pathways; inhibited apoptosis	Aydos et al. (2014)
	Wistar rat	Doxorubicin-induced TT model	Dosage:0.5 mg/kg (First week)→ 1 mg/kg (Second and third weeks)	Therapeutic intervention (Immediately administer after reperfusion/p.r.)	21 days after reperfusion	Enhanced antioxidant capacity; reduced inflammation; improved physiological function	Salem et al. (2017)
	Wistar rat	TT I/R model	Concentration:50 μg/mL Dosage:4 mg/kg	Therapeutic intervention (15 min before reperfusion/i.p.)	7 days after reperfusion	Enhanced antioxidant capacity; improved histopathology and physiological function	Ekici et al. (2012)
	SD rat	TT I/R model	Dosage:1 mg/kg	Preconditioning (Immediately administer beforeischemia/i.p.)	24 h after reperfusion	Reduced IMA; enhanced antioxidant capacity; improved histopathology	Tusat et al. (2017)
Brain	C57BL/6 mice; SH-SY5Y cells	tMCAO model; H₂O₂-Induced cell damage model	Concentration:20 μg/mL Dosage:1.5 mg/kg	Therapeutic intervention (Immediately administer after reperfusion/i.p.)	24 h after reperfusion	Upregulated PPARγ or Nrf2; inhibited ROS-Ca²⁺ vicious cycle; inhibited parthanatos	Li et al. (2024)
	SD rat	MCAO/R model	Concentration:40 μg/mL Dosage:2.5 mL/kg/d	Preconditioning (5 days before ischemia/i.p.)	24 h after reperfusion	Activated the Nrf2/Slc7a11/GPX4 pathway ; inhibited ferroptosis;	Zhu et al. (2024)
​	SD rat	MCAO/R model	Dosage:2 mg/kg	Therapeutic intervention (Immediately after ischemia/i.p.)	5 days after reperfusion	Inhibited NF-κB pathway, autophagy and neuronal apoptosis; enhanced antioxidant capacity	Zhu et al. (2022)
	Rat brain slice	OGD/R model	Concentration:20–160 μg/mL	Therapeutic intervention (30 min after ischemia/Ozonated ACSF)	1.5 h after reperfusion	Reduce excitotoxicity and cellular damage; enhanced antioxidant capacity	Frosini et al. (2012)
	Rat brain slice	OGD/R model	Concentration:10–50 μg/mL	Therapeutic intervention (30 min after ischemia/Ozonated ACSF)	1.5 h after reperfusion	Inhibited LDH and glutamate	Shi et al. (2014)
	SH-SY5Y cell	OGD/R model	Concentration:20 μg/mL	Preconditioning (2 h before ischemia/Ozonated Culture Medium)	Immediately after reperfusion	Reduced cyt-c release; inhibited the mitochondrial-mediated apoptosis and caspase pathways; enhanced the Bcl-2/Bax ratio	Zhu et al. (2020)
Lung	SD rat	Left lung I/R model	Dosage:100 μg/kg	Preconditioning (10 days before ischemia/i.p.)	1 day after reperfusion	Activated the Nrf2/Slc7a11/GPX4 pathway; enhanced antioxidant capacity; inhibited NLRP3 activation; anti-inflammatory (reduced inflammatory cell infiltration); inhibit apoptosis	Wang L. et al. (2018)
Cochlea	Guinea pig	Bilateral vertebral artery and vein ligation-induced cochlear ischemia-reperfusion model	Concentration:60 μg/mL Dosage:1 mg/kg	Preconditioning (7 days before ischemia/i.p.)	2 h after reperfusion	Enhanced antioxidant capacity	Onal et al. (2017)
Retina	Wistar rat	130 mmHg intraocular pressure-induced retinal ischemia-reperfusion model	Concentration:60 μg/mL Dosage:1 mg/kg	Preconditioning (7 days before ischemia/i.p.)	2 h after reperfusion	Enhanced antioxidant capacity	Kal et al. (2017)
Skin flap	Wistar rat	Axillary artery Ligation-induced muscle flap ischemia-reperfusion model	Concentration:60 μg/mL Dosage:1 mg/kg	Therapeutic intervention (Immediately administer after reperfusion/i.p.)	7 days after reperfusion	Enhanced antioxidant capacity	Elsurer et al. (2020)
Muscle	Wistar rat	IA I/R model	Concentration:60 μg/mL Dosage:0.7 mg/kg	Preconditioning (72 h before ischemia/i.p.)	22 h after reperfusion	Reduced iNOS expression; enhanced antioxidant capacity; reduce nitrification stress	Koca et al. (2010)
	Wistar rat	FA I/R model	Concentration:60 μg/mL Dosage:0.7 mg/kg	Preconditioning (72 h before ischemia/i.p.)	22 h after reperfusion	Enhanced antioxidant capacity; anti-inflammatory	Ozkan et al. (2015)
	Wistar rat	IA I/R model	Concentration:60 μg/mL Dosage:1 mg/kg	Therapeutic intervention (14 days before ischemia/i.p.)	14 days after reperfusion	Enhanced antioxidant capacity; reduced iNOS expression; enhanced PCNA expression; inhibited apoptosis and inflammation	Gawish et al. (2022)
	Wistar rat	IA I/R model	Concentration:60 μg/mL Dosage:0.7 mg/kg	Preconditioning (72 h before ischemia/i.p.)	22 h after reperfusion	Enhanced antioxidant capacity	Koca et al. (2010)

OT, ovarian torsion; TT, testicular torsion; tMCAO, transient middle cerebral artery occlusion; MCAO, middle cerebral artery occlusion; MCAO/R, Middle Cerebral Artery Occlusion/Reperfusion; IMA, ischemia-modified Albumin; ACSF, artificial cerebrospinal fluid; IA, ileal artery; FA, femoral artery.

### Heart

Cardiovascular disease is the leading cause of mortality and morbidity worldwide, and ischemic heart disease is a significant contributor to the high mortality rates (Roth et al., 2020). Timely restoration of the myocardial blood flow is a critical therapeutic approach to salvage ischemic myocardial tissue. However, cardiac reperfusion can cause further damage to myocardial cells and even induce secondary cardiac dysfunction, known as myocardial I/R injury. Coronary artery disease, thromboembolic cardiomyopathy, cardiac surgery, and cardiogenic shock frequently result in myocardial I/R injury.

Several clinical studies have shown that low-dose ozone (<50 μg/mL) significantly improves cardiac function and antioxidant status in patients (Buyuklu et al., 2017; Pandolfi et al., 2024). The controlled oxidative stress induced by ozone exposure may confer protection against pathophysiological alterations caused by myocardial I/R (Koçyiğit et al., 2018). In some studies, ozone pre-treatment significantly reduced the percentage of ischemic myocardial mass relative to left ventricular mass (ICS/LV), the percentage of necrotic myocardial mass relative to ischemic myocardial mass (IFS/ICS), and cardiac troponin I (cTnI) levels compared to those in the untreated control group (Koçyiğit et al., 2018). Di Filippo et al. reported that intraperitoneal administration of ozone 1 h before myocardial I/R in a rat model prevented tissue injury by modulating oxidative, inflammatory, immune, and apoptotic responses (Di Filippo et al., 2008). Furthermore, Meng et al. found that ozone pre-conditioning protected myocardial tissue against I/R injury by activating the Nrf2 antioxidant pathway and reducing oxidative stress (Meng et al., 2017). Another study demonstrated that ozone pretreatment significantly mitigated myocardial I/R injury by inhibiting NLRP3 inflammasome activation and reducing caspase-1-mediated IL-1β/IL-18 release, and the effects were more pronounced at ozone concentrations below 40 μg/mL (Xu et al., 2025). As a core regulator of oxygen adaptation, hypoxia-inducible factor-1α (HIF-1α) is also modulated by ozone pretreatment. Ozone pretreatment exerts a protective effect by upregulating HIF-1 in the rabbit heart against ischemia-reperfusion injury (Wang R. et al., 2022). Furthermore, ozone has been shown to protect against myocardial damage induced by aortic I/R and streptozotocin-induced myocardial I/R in diabetic rats (Burak Gulcan et al., 2024; Gülcan et al., 2024). Heat shock protein 70 (HSP70) plays a crucial role in protein metabolism by promoting the folding of newly synthesized peptides and removing denatured proteins (Chen et al., 2013). As a conserved protein, HSP70 inhibits cell death by suppressing ROS production and inhibiting caspase-3 and caspase-7 activation (Komarova et al., 2004). Ozone-protected cardiomyocytes from I/R injury-induced death by upregulating HSP70 expression in a dose-dependent manner through activation of the JAK2/STAT3 pathway (Yu et al., 2021). Meng et al. showed that ozone mitigated mitochondrial damage and apoptosis in a myocardial I/R injury model by inhibiting Cyt-c release from mitochondria into the cytoplasm and suppressing the caspase pathway (Meng et al., 2017). The miR-200 family plays a critical role in cardiovascular diseases (Magenta et al., 2017). MiR-200C upregulation in rat I/R models is associated with increased myocardial infarction area, and its suppression protects against hypoxia-induced cardiomyocyte apoptosis (Chen Z. et al., 2017). Ozone treatment has been shown to inhibit post-ischemic myocardial cell death by downregulating FOXO3, a downstream target of miR-200C (Zhang et al., 2022). Autophagy is a self-catabolic process that plays a vital role in cellular survival and protects tissues and organs during ischemia (Shi et al., 2019; Dugbartey, 2024). However, excessive autophagy induces cell death following reperfusion (Filomeni et al., 2015). Ozone treatment has been shown to downregulate autophagy proteins, increase the Bcl-2/BAX ratio, and decrease IκB-caspase-3 activation in cardiac tissues during I/R, indicating that it can protect the myocardium from I/R injury by inhibiting excessive autophagy (Xu et al., 2021). Consistent with this, Ding et al. showed that ozone reduced oxidative stress responses and ferroptosis by promoting nuclear translocation of Nrf2 and upregulating Slc7a11 and GPX4 (Ding et al., 2023). Ferroptosis is a form of cell death caused by excessive iron accumulation resulting from lipid peroxidation (Mou et al., 2019). Meanwhile, cuproptosis, which is a form of cell death characterized by disruption of the core metabolic pathway, the mitochondrial tricarboxylic acid (TCA) cycle, has been demonstrated to play a significant role in ischemia-reperfusion injury (Li S.-R. et al., 2022). It is still unclear whether ozone’s protective effect against ischemia-reperfusion injury also acts by inhibiting cuproptosis (Zhang et al., 2017; Chen et al., 2024). Elevated eNOS also plays a crucial role in the defense against I/R injury (Zhang et al., 2017). Di Filippo et al. found that ozone protected the heart from acute myocardial infarction by locally increasing eNOS activity and recruiting endothelial progenitor cells (Di Filippo et al., 2010b). Di Filippo et al. proposed that a mixture of oxygen and ozone can exert dual cardioprotective effects by restoring ion balance and maintaining myocardial electrical integrity (Di Filippo et al., 2010a). Furthermore, the restorative effect of ozone on ionic balance, when combined with lidocaine, synergized to mitigate myocardial I/R injury-induced arrhythmia. This suggests potential effects of ozone on Na^+^ channels, which will need to be experimentally validated. Myocardial I/R injury during heart transplantation is unavoidable. One study showed that ozone pretreatment significantly reduced immune rejection after heart transplant in a rat model and even eliminated acute rejection, resulting in prolonged survival of the transplanted heart (Stadlbauer et al., 2008). In another study, suffusing ozonated water during reperfusion in a rat model mitigated myocardial injury (Merin et al., 2007). Pre-treatment with higher doses of ozone (<1 mg/kg) was effective against the pathophysiological changes associated with I/R injury, indicating that the cardioprotective action of ozone is dose-dependent (Ahmed et al., 2012). Furthermore, increasing the final dose of ozone rather than the overall concentration achieved superior efficacy against myocardial I/R injury (Wang R. et al., 2022). In fact, a “therapeutic window” of 10–80 μg/mL ozone can produce therapeutic effects without toxicity by ensuring mild and precise oxidative stress (Sagai and Bocci, 2011). Ozone administration via intestinal insufflation at specific doses has been shown to prevent myocardial I/R injury, providing a reference for future clinical application. Autologous major blood ozonation has also demonstrated cardioprotective effects in some studies; however, due to safety concerns and other factors, it is not currently recommended.

### Liver

Liver surgery, including hepatectomy, is a crucial therapeutic approach for liver diseases such as cirrhosis and hepatocellular carcinoma, and liver transplantation remains the preferred treatment for patients with end-stage liver disease (Sykes and Sachs, 2022). Prolonged ischemia during these surgical procedures induces liver damage, while postoperative reperfusion further impairs hepatic and vascular endothelial functions, and adversely affects postoperative recovery (Simillis et al., 2016). Numerous studies have demonstrated that ozone treatment can protect against hepatic I/R injury by regulating redo x balance (Peralta et al., 2000; Ajamieh et al., 2004). Peralta et al. observed that pre-treatment with ozone reduced levels of lactate, aspartate aminotransferase, and alanine transaminase, maintained ionic equilibrium in hepatocytes, and mitigated tissue injury in a mouse model of hepatic I/R (Peralta et al., 1999). Concurrently, ozone increased SOD activity and GSH levels and reduced H_2_O_2_ levels following hepatic I/R injury, thus mitigating oxidative damage (Peralta et al., 1999). Mn-SOD serves as the first line of defense against O_2_^−^• production. Ozone pretreatment mitigated I/R injury-mediated oxidative stress by activating Mn-SOD isoforms and preventing GSH depletion (Ajamieh et al., 2005). NO also plays a significant role in hepatic I/R injury and protection (Zhang et al., 2017). Peroxynitrite (ONOO^−^), a powerful oxidant formed during the reaction of NO and O_2_^−^•, exerts significant cytotoxicity during I/R injury (Lefer et al., 1997; Liu et al., 1998). Nevertheless, the anti-inflammatory action of NO may outweigh its cytotoxic effects (Liu et al., 1998; Ajamieh et al., 2005). Furthermore, neuronal NOS (nNOS), inducible NOS (iNOS), and eNOS have distinct roles in I/R injury (Liu et al., 1998; Ajamieh et al., 2005). In a study conducted by Ajamieh et al., low-dose ozone activated NOS-encoding genes, alleviating hepatic I/R injury (Ajamieh et al., 2004). Furthermore, ozone pretreatment also increased adenosine levels, which prevented downregulation of eNOS and increased NO production (Serracino-Inglott et al., 2002; Ajamieh et al., 2004). Similar to the findings of Peralta et al., ozone pretreatment protected against hepatic I/R injury by maintaining cellular redox balance (Peralta et al., 1999; Ajamieh et al., 2004). Ozone-induced elevation of adenosine, a key component of vascular homeostasis, may also inhibit pro-inflammatory nuclear transcription factors and exert a protective effect against hepatic I/R injury (Peralta et al., 2000; Ajamieh et al., 2004). Kupffer cells are activated in the early stages following reperfusion in ischemic areas and produce excessive amounts of ROS and pro-inflammatory cytokines (Nakamitsu et al., 2001). Ozone treatment may prevent Kupffer cell activation through its preadaptation mechanism and reduce or prevent increases in ROS precursors and inflammatory factors such as TNF-α, thereby mitigating liver injury following I/R (Peralta et al., 2000; Ajamieh et al., 2002). Ischemic pre-conditioning involves the induction of organ stress, which enhances endogenous defense systems and confers greater tolerance to subsequent I/R injury (Peralta et al., 2002). It represents an effective clinical strategy for preventing hepatic I/R injury. Ajamieh et al. demonstrated comparable protective effects of ischemic pre-conditioning and ozone pretreatment against hepatic I/R injury, raising the possibility of similar biochemical protective mechanisms (Ajamieh et al., 2002). However, histological observations indicated superior therapeutic effects of medical ozone treatment compared to ischemic pre-conditioning (Ajamieh et al., 2003). This finding suggests that, in protecting against ischemia-reperfusion injury, ozone pretreatment may possess additional, and possibly superior, protective mechanisms beyond those shared with ischemia pretreatment, which warrant further exploration. Adenosine plays a vital role in regulating smooth muscle tone by inducing vascular smooth muscle relaxation through cAMP-mediated cascades. Furthermore, adenosine exerts protective effects against hepatic I/R injury by preventing eNOS downregulation in hepatic sinusoidal cells, which in turn suppresses free radical production, leukocyte adhesion, and neutrophil superoxide generation, and inhibits the pro-inflammatory nuclear transcription factors (Li et al., 2000; Ramkumar et al., 2001; Serracino-Inglott et al., 2002). Studies have found that ozone can increase adenosine accumulation by inhibiting adenosine deaminase (ADA) activity. Combined with ozone’s reduction in hypoxanthine and xanthine accumulation, it can be inferred that ozone may block the xanthine/xanthine oxidase pathway to generate ROS, thereby decreasing post-reperfusion ROS production and protecting against hepatic I/R injury (Xia et al., 1996; Peralta et al., 2000). The adenosine receptors include A1 (A1AR), A2 (A2AR), and A3 (A3AR) subtypes. Olga S. et al. demonstrated that ozone pre-treatment exerted hepatoprotective effects against I/R injury by activating A1AR (León Fernández et al., 2007). On the other hand, A2AR is associated with ischemic pre-conditioning (Carini and Albano, 2003). Both ischemic and ozone pre-conditioning generate adenosine and NO by regulating NF-κB (p65 subunit) and HSP-70 (Peralta et al., 2000; León Fernández et al., 2007). Studies show that activation of p38 MAPK may be responsible for enhancing the resistance of pre-conditioned livers to oxidative stress (Lee et al., 2003). Ozone pre-treatment also prevents sustained NF-κB activation during subsequent reperfusion, reduces TNF-α levels, upregulates HSP70, and inhibits apoptotic pathways (León Fernández et al., 2007). The beneficial effects of ozone have also been demonstrated in a rat model of liver regeneration following partial resection (Gultekin et al., 2013). Gultekin et al. administered intraperitoneal ozone injections to rats before hepatectomy surgery. Postoperative changes in the levels of serum transaminases, TNF-α, and IL-6, and in hepatocyte morphology and liver histopathology indicated that ozone pretreatment mitigated liver injury and promoted hepatocyte regeneration (Gultekin et al., 2013). Furthermore, the protective effect of ozone was linked to reduced ROS levels, TNF-α-mediated NF-κB activation, and IL-6 upregulation (Zamora et al., 2005).

### Kidney

Renal I/R injury is a common cause of acute kidney injury (AKI), often resulting from hypovolemia, septic shock, accidental or iatrogenic trauma, cardiovascular surgery, and renal surgery, and with no truly effective therapeutic solution (Wang et al., 2014; Han and Lee, 2019; Ren et al., 2020). Ozone treatment significantly reduced oxidative stress in the kidney after reperfusion, increased antioxidant enzyme activity, and alleviated histopathological damage due to renal I/R injury (Oztosun et al., 2012; Wang et al., 2014). The protective effect of ozone on renal I/R is closely linked to its anti-inflammatory, antioxidant, and anti-apoptotic functions. Ozone pretreatment reduced markers of oxidative stress and inflammation, including myeloperoxidase activity, IL-1β, TNF-α, and ICAM-1, while increasing the levels of the anti-apoptotic protein Bcl-2 and inhibiting Bax translocation to the mitochondria (Chen et al., 2008a; Xing et al., 2015). This, in turn, inhibited mitochondrial Cyt c release and caspase-3 activation, eventually reducing I/R-induced apoptosis (Cai et al., 2020). The anti-apoptotic effect of ozone was also mediated via the inactivation of the MAPK pathway (Wang L. et al., 2018). In addition, ozone mitigated I/R injury-related oxidative stress by reversing the changes in SOD and MDA levels. Furthermore, ozone pretreatment significantly downregulated the TLR4/NF-κB pathway and reduced the levels of inflammatory factors such as TNF-α, IL-1, IL-6, intercellular adhesion molecule-1 (ICAM-1), and monocyte chemotactic protein-1 (MCP-1) (Xing et al., 2015). Another study showed that ozone pretreatment protected against I/R injury-induced renal dysfunction by increasing eNOS and iNOS expression, thereby mediating endogenous NO production, while simultaneously inhibiting the increase in renal tissue endothelin-1 (ET-1) (Chen et al., 2008c). Wang et al. revealed that both ischemia pre-treatment and ozone pre-treatment suppressed the I/R-induced elevation in renal caspase-1, caspase-11, IL-1β, and IL-18 levels, thereby preventing renal injury and pyroptosis (Wang et al., 2019). The combination of both approaches yielded superior outcomes, suggesting distinct mechanisms of action; nevertheless, NO appears to be a common mediator (Chen et al., 2008c). High-dose ozone infusion using autologous blood effectively mitigates renal tissue injury caused by I/R through anti-inflammatory and antioxidant effects (Foglieni et al., 2011; Sancak et al., 2016). Foglieni et al. observed that high-dose autologous ozone blood pretreatment limited endothelial injury induced by ischemia and transient reperfusion in a rat model of unilateral nephrectomy and protected renal tissues (Foglieni et al., 2011). Research has shown that this effect was unrelated to changes in plasma NO levels and was linked to increased renal β-NADPH-xanthine oxidoreductase activity and mitochondrial protection (Foglieni et al., 2011). Although ozone autologous blood therapy is an effective treatment, its safety remains questionable. Furthermore, studies conducted by Wang et al. and Jiang et al. demonstrated that ozone pre-treatment not only reduced oxidative stress and inflammatory responses induced by renal I/R injury but also decreased the expression levels of TGF-β1, α-smooth muscle actin (α-SMA), and Smad7, thereby inhibiting renal fibrosis that may occur in the late stage of I/R (Wang et al., 2014; Jiang et al., 2020). Thus, medical ozone treatment is a promising approach to prevent renal fibrosis and long-term renal I/R-induced damage.

The short- and long-term protective effects of ozone against renal I/R injury have been demonstrated across renal function, histomorphology, and molecular biology. However, the underlying mechanisms remain unclear and require further investigation. In addition, a safe and reliable protocol for ozone administration must be established for eventual clinical applications.

### Intestine

Gastrointestinal ischemia can be caused by mesenteric embolism, intestinal volvulus, intussusception, and intestinal transplantation surgery. Intestinal reperfusion and subsequent I/R injury in the gastrointestinal tract can lead to multiple organ failure, intestinal atrophy, sepsis, and vascular protein and fluid leakage, constituting a critical condition with high morbidity and mortality (Isik et al., 2015; Bertoni et al., 2018). There are currently no effective measures or drugs for mitigating intestinal I/R injury (Li G. et al., 2022). Prophylactic ozone pretreatment in intestinal I/R models has been shown to increase SOD, GPX, and CAT levels, enhance total antioxidant status (TAS), and simultaneously reduce inflammation and inflammatory cell infiltration (Altinel et al., 2011; Onal et al., 2015). Furthermore, histopathological evaluations indicate that ozone pretreatment can prevent I/R-induced mucosal injury in the gut (Onal et al., 2015). However, Günal et al. found that a single dose of ozone administered 20 min before intestinal I/R did not reduce oxidative stress or improve ischemic injury in a rat model (Dere Günal, 2020). These contradictory findings may be related to factors such as ozone dosage (single vs. repeated doses) or the route and timing of administration (Erginel, 2023). Erginel et al. demonstrated that increasing the ozone dose to 1 mg/kg effectively mitigated intestinal I/R injury, primarily by enhancing antioxidant enzyme activity (e.g., GSH) and inhibiting lipid peroxidation (reduction in MDA levels)(Dere Günal, 2020; Erginel, 2023). Thus, insufficient ozone dosage or differences in detection parameters may affect the outcomes. Furthermore, ozone has demonstrated superior therapeutic efficacy against intestinal I/R injury compared with naringenin and trimetazidine (Isik et al., 2015; Demiral et al., 2024). In a study by Haj et al., ozone treatment significantly reduced intestinal cell apoptosis after I/R, as indicated by downregulation of caspase-3 and a decreased Bax/Bcl-2 ratio, and increased intestinal epithelial cell proliferation by upregulating phosphorylated ERK (p-ERK) expression (Haj et al., 2014). The ERK pathway is one of the MAPK signaling pathways and controls cell proliferation, mitosis, differentiation, and apoptosis in response to cytokines or growth factors (Zhou et al., 2018). Based on promising outcomes in experimental intestinal I/R injury models, ozone pretreatment should be further explored as an effective therapeutic approach to manage intestinal ischemic injury and restore intestinal function post-reperfusion. However, the specific mechanisms underlying the protective effects of ozone against intestinal I/R injury require further investigation.

### Gonads

Ovarian torsion is a rare surgical emergency in pediatric patients, and its treatment remains controversial (Galinier et al., 2009). Currently, patients undergo adnexal detorsion to preserve the ovaries. However, postoperative I/R injury to the ovaries may lead to severe local and systemic consequences. The protective effects of low-dose ozone on ovarian I/R injury were evaluated in a rat model of ovarian torsion I/R. Intraperitoneal administration of a mixture of oxygen and ozone to rats 10 min before I/R significantly improved congestion, hemorrhage, interstitial edema (I.E.,), and polymorphonuclear neutrophil infiltration, and also reduced MDA, NO, and total sulfhydryl (t-SH) levels (Aslan et al., 2012). Various antioxidant and anti-inflammatory therapies have been explored to protect ovarian function and protect against postoperative I/R injury. Ozone has been experimentally demonstrated to sustain ovarian function and reserve following surgery. In one study, administration of an ozone/oxygen mixture 15 min before reperfusion and daily for 7 days postoperatively in an ovarian torsion model, potentially through anti-inflammatory and antioxidant mechanisms (Süzen Çaypınar et al., 2022). Sayar et al. observed that the use of ozone, ellagic acid, or their combination significantly increased antioxidant enzyme activity and reduced MDA levels in a rat model of ovarian I/R, resulting in better tissue preservation (Sayar et al., 2016). However, histopathological indices, such as ellagic acid, appeared more effective than ozone (Sayar et al., 2016).

Testicular torsion (TT) is commonly observed in neonates, children, and adolescents and results from impaired blood flow to the testis and epididymis following rotation of the spermatic cord and vessels (Yang et al., 2011). Prolonged ischemia can lead to organ dysfunction. Testicular salvage requires early restoration of blood flow, but subsequent I/R injury poses a significant challenge (Mete et al., 2017; Kylat and Ahmed, 2022). Tusat et al. demonstrated that medical ozone treatment reduced levels of ischemia-modified albumin (IMA), total antioxidant status (TAS), total oxidative status (TOS), oxidative stress index (OSI), and histopathological scores in a testicular I/R model (Tusat et al., 2017). Salem et al. also reported that medical ozone treatment mitigated doxorubicin-induced testicular toxicity in an experimental rat model by reducing oxidative stress and NO levels (Salem et al., 2017). Melatonin can directly scavenge ROS and inhibit ROS production while increasing the production and activity of cellular antioxidant enzymes (Chrustek and Olszewska-Słonina, 2021). The effects of melatonin and ozone on unilateral testicular I/R injury were compared. While medical ozone treatment reversed MDA and GSH levels, melatonin treatment substantially reduced I/R injury in the testis, as assessed by the Johnsen score (Ekici et al., 2012).

Furthermore, melatonin also restored function in the contralateral testis. The two treatments had different effects on NO levels, which may offer new insights into the mechanisms and therapeutic applications of ozone for I/R injury. Aydos et al. found that medical ozone treatment and taurine prevented testicular I/R injury in a rat model of TT by reducing apoptosis and eNOS expression, while increasing CAT activity (without altering antioxidant enzyme levels) (Aydos et al., 2014). Pre-reperfusion ozone treatment may offer a way to prevent germ cell degeneration and associated infertility caused by testicular torsion. Ozone and taurine treatment reduced the levels of TNFR1, caspase 3, and caspase eight induced by testicular I/R injury, but did not affect Cyt c and TNF-α (Aydos et al., 2014). This suggests that the extrinsic apoptosis pathway may play a key role in testicular I/R injury. Ozone achieved better therapeutic effects in an experimental model of distal colitis than hyperbaric oxygen (Altinel et al., 2011). In the testicular I/R model, however, ozone and hyperbaric oxygen achieved similar histopathological outcomes, although hyperbaric oxygen showed superior antioxidant effects (Karlı, 2023). Nevertheless, both therapies provided protective effects against testicular I/R injury. Mete et al. compared the effects of local and systemic testicular administration of ozone and found that intra-testicular ozone treatment yielded superior efficacy compared to systemic treatment via intraperitoneal administration (Mete et al., 2017). This provides a novel approach for future clinical applications aimed at preventing I/R injury following TT surgery.

Although the precise mechanism underlying the protective effects of ozone on the gonads remains to be elucidated, the clear benefits of medical ozone treatment for I/R injury caused by ovarian torsion, TT, and similar conditions offer an alternative for managing related diseases (Naserzadeh et al., 2019). Furthermore, ozone may serve as a therapeutic approach for the pre-degenerative or pre-necrotic stage of I/R-induced cellular changes in reproductive organs, offering new hope for preserving reproductive function.

### Others

Ischemic stroke is a leading cause of death and disability among adults worldwide (Gorelick, 2019). Cerebral blood flow is interrupted following a stroke, depriving the brain of oxygen and nutrients. Although reperfusion occurs after thrombolysis, the restored blood flow can cause further brain damage. Given its anti-inflammatory, antioxidant, anti-apoptotic, and autophagy-promoting properties, ozone has significant potential to delay neurodegeneration (Scassellati et al., 2020). Infusion of 120 μg/mL ozone protected rat brain slices from oxygen-glucose deprivation and reperfusion, antagonized glutamate and lactate dehydrogenase release, reduced tissue edema, and exhibited enhanced efficacy in artificial cerebrospinal fluid containing human albumin (Frosini et al., 2012). Shi et al. replicated these protective effects of ozone on isolated rat brain slices and identified 40 μg/ml as the optimal dose (Shi et al., 2014). Thus, a lower ozone dosage may yield superior outcomes. Furthermore, ozone treatment exerted neuroprotective effects in a rat model of cerebral I/R injury by increasing NRF2 nuclear translocation and SLC7A11/GPX4 expression, mitigating oxidative stress, and reducing ferroptosis via the NRF2/SLC7A11/GPX4 pathway (Zhu et al., 2024). Li et al. found that ozone activates the PPARγ/Nrf2 pathway, inhibits ROS bursts and Ca^2+^ overload, and breaks the ROS-Ca^2+^ vicious cycle, thereby reducing parthanatos after stroke (Li et al., 2024). In a case report by Clavo et al., medical ozone treatment alleviated cerebral I/R injury by mitigating inflammatory responses and increasing blood flow (Clavo et al., 2011). *In vitro* studies using SH-SY5Y cells have confirmed that ozone inactivated the caspase-3 apoptosis cascade by inhibiting mitochondrial Cyt c release and increasing the Bcl-2/Bax ratio (Cai et al., 2020). These studies collectively support the application of ozone for cerebral ischemic diseases, though its precise mechanisms remain to be elucidated. In addition, the optimal concentration, dosage selection, and administration methods also warrant further investigation.

Pulmonary I/R injury can potentially lead to severe outcomes. However, effective management strategies for these complications remain elusive (Eltzschig and Eckle, 2011). Previous studies have demonstrated that administration of a hyperoxic solution protected the endogenous antioxidant system in rabbits with intestinal I/R injury and reduced lipid peroxidation (Gao et al., 2008). Given its potent oxidant properties, direct inhalation of ozone can cause severe damage to lungs lacking antioxidant capacity. However, altering the ozone delivery method achieved therapeutic effects similar to those of hyperoxic solution pre-treatment, and protected against pulmonary I/R injury. Wang et al. reported that ozone pretreatment significantly mitigated mitochondrial damage in a rat model of pulmonary I/R injury by activating the Nrf2 pathway, suppressing the NLRP3 inflammasome, and reducing apoptosis (Wang Z. et al., 2018).

During surgical procedures, particularly limb and orthopedic surgeries, tourniquets are frequently applied to reduce distal blood flow, creating ischemic or even avascular zones to minimize intraoperative bleeding. Tourniquet removal subsequently induces I/R injury, which can cause acute renal failure, respiratory failure, cardiac dysfunction, and even death (Ozyurt et al., 2007). Hypothermia can counteract I/R injury in skeletal muscles by reducing metabolic rate, thereby decreasing I/R-induced tissue edema, acidosis, oxygen consumption, and muscle infarction (Eskla et al., 2022). Both hypothermia and ozone pretreatment mitigated tourniquet-induced I/R injury in rat skeletal muscle by reducing oxidative and nitrosative stress parameters (nitrite levels) while enhancing antioxidant enzymes (Ozkan et al., 2015). However, the effect of ozone was significantly weaker than that of hypothermia treatment, and no synergism was observed between the two approaches. This discrepancy may stem from shared mechanisms underlying ozone and hypothermia in protecting skeletal muscle against I/R injury. On the other hand, both ozone and hyperbaric oxygen pre-treatment reduced oxidative and nitrosative stress and iNOS staining scores in ischemic-reperfused skeletal muscle. No significant differences were observed (Koca et al., 2020). Erythropoietin (EPO) exerts anti-inflammatory, antioxidant, and anti-apoptotic effects by reducing oxidative stress, enhancing enzymatic antioxidant activity, and scavenging free radicals (Golmohammadi et al., 2020). Gawish et al. demonstrated that both ozone and EPO protected against skeletal muscle I/R injury (Gawish et al., 2022). In addition, ozone or hyperbaric oxygen pre-treatment has been shown to reduce levels of MDA and protein carbonyls and to increase SOD and GPX levels in bone tissue following I/R injury, which may have clinical significance for bone recovery after fractures (Koca et al., 2010). These studies collectively demonstrate that medical ozone treatment reduces I/R injury in skeletal muscle and bone tissue, indicating significant potential in trauma emergency care, orthopedics, and extremity surgeries involving tourniquet application.

Consistent with previous research, ozone reduced free radical-mediated tissue I/R injury and oxidative stress after flap surgery by promoting cell proliferation and reducing apoptosis (Elsurer et al., 2020). This may enhance the success rate of flap surgery and reduce the likelihood of secondary procedures. The cochlea exhibits high sensitivity to blood supply and oxygenation, and any interruption in blood flow can cause severe functional damage (Kim et al., 1999). Cochlear I/R injury is considered one of the most significant causes of idiopathic sudden sensorineural hearing loss in humans, for which effective treatments remain limited (Tabuchi et al., 2010). Ozone treatment before cochlear ischemia reduced apoptosis, enhanced antioxidant capacity, mitigated oxidative stress induced by transient cochlear I/R, and alleviated morphological damage, suggesting potential clinical applications of ozone for treating sudden deafness (Onal et al., 2017). The retina is also a metabolically active tissue and, therefore, susceptible to disruptions in blood and oxygen supply. Retinal I/R injury can lead to various ocular diseases and even result in retinal dysfunction and vision loss by producing massive amounts of ROS and increasing apoptosis (Kim et al., 2013). Ozone administration before retinal I/R effectively reduced ROS levels, enhanced the retina’s antioxidant capacity, diminished inflammatory responses, and decreased retinal ganglion cell death, thereby mitigating retinal I/R injury (Kal et al., 2017).

## Discussion

Ever since ozone was first used in medicine over a century ago, there have been consistent efforts to expand its biomedical applications. Studies show that low-dose ozone administration mitigates inflammation and oxidative stress, improves microcirculation, and inhibits apoptosis and ferroptosis, thereby exerting organ-protective effects (Bocci et al., 2009). At the molecular level, ozone modulates the caspase, NRF2/SLC7A11/GPX4, JAK2/STAT3, TNF-α, NF-κB, and other signaling pathways (Xing et al., 2015; Yu et al., 2021; Ding et al., 2023; Xu et al., 2025). A greater understanding of these mechanisms can offer novel insights into the potential clinical applications of ozone, such as organ recovery after ischemic injury. While ozone pre-treatment has been shown to exert short- or long-term protective effects against I/R injury in animal models, the clinical safety and efficacy of medical ozone treatment remain to be ascertained. Furthermore, the protective effect of ozone against I/R injury has been experimentally demonstrated in multiple organs, but relatively little is known regarding its impact on the nervous and respiratory systems. The unique physicochemical properties of ozone pose challenges for its widespread clinical application. First, the difficulty in obtaining ozone and its inherent instability necessitate the development of safer, more stable methods for its production, long-term storage, detection, and application. Second, given ozone’s potent oxidative properties, patients may experience significant distress during treatment and potentially greater harm following I/R injury if the gas is not administered properly. Thus, identifying safe and rational delivery methods is crucial. Third, the therapeutic efficacy of ozone is dependent on its concentration and dosage, and optimal effects are observed within specific concentration-dose ranges, indicating a delicate balance between efficacy and safety. Therefore, further trials are needed to determine appropriate ozone dosages for various clinical scenarios.

Pre-treatment with a controlled dose of ozone protects tissues and organs from I/R injury by inducing the body’s inherent defense systems (Erginel, 2023). Ozone pre-treatment exhibits effects similar to ischemic pre-conditioning and hyperbaric oxygen pre-treatment (Ajamieh et al., 2002; Chen et al., 2008c). The non-invasive nature of ozone offers an advantage over ischemia, which is difficult to implement clinically. Furthermore, both hyperbaric oxygen and ozone pretreatment, as representative examples of hormesis, share numerous mechanisms of action. The core mechanism for enhancing antioxidant capacity and mitigating ischemia-reperfusion injury involves regulating oxidative stress through activating the Nrf2 pathway (Yin et al., 2015; Hentia et al., 2018; Cisterna et al., 2020; Zhu et al., 2024). Additionally, these therapies play crucial roles in protecting mitochondrial structure and function. They stabilize mitochondrial membrane potential, mitigate oxidative stress and mitochondrial damage-induced cell death, and reduce cell death by increasing the Bcl-2/Bax ratio and decreasing caspase-3 activation (Hentia et al., 2018; Cai et al., 2020; Wang et al., 2021; Xu et al., 2012). Both hyperbaric oxygen and ozone pretreatment can mitigate inflammatory responses by inhibiting the NF-κB pathway (Chen et al., 2008b; Xing et al., 2015; Hentia et al., 2018). Meanwhile, both can improve microcirculation and regulate immune cell function to protect against ischemia-reperfusion injury (Chen et al., 2008b; Hentia et al., 2018). Despite similar mechanisms, ozone, a more “aggressive” stimulus than hyperbaric oxygen, interacts with endogenous second messengers, such as hydrogen peroxide and lipid oxidation products, to activate MAPK and AMPK pathways against ischemia-reperfusion injury (Zhang et al., 2017; Wang L. et al., 2018). Moreover, ozone-induced HSP70 enhances protection against ischemia-reperfusion injury (Yu et al., 2021). Concurrently, ozone reduces oxygen affinity of hemoglobin, facilitating oxygen release from oxyhemoglobin in tissues and directly improving oxygen supply to ischemic areas, which explains its superior protective efficacy against ischemia-reperfusion injury (Bocci et al., 2011; Zinchuk and Biletskaya, 2023). Both hyperbaric oxygen pretreatment and ozone pretreatment suppress excessive autophagy to counteract ischemia-reperfusion injury. However, hyperbaric oxygen pretreatment can promote protective autophagy flux under certain conditions (Wang Y.-C. et al., 2010; Filomeni et al., 2015; Chen C. et al., 2017; Xu et al., 2021). This promotion of protective autophagy flux may also contribute to ozone pretreatment’s protective effects against ischemia-reperfusion injury, though further research is needed to confirm this (Wang Y.-C. et al., 2010; Chen C. et al., 2017). Although the protective effect of ozone on mitochondria has been demonstrated, its specific mechanism remains unclear. Hyperbaric oxygen pretreatment promotes mitochondrial biogenesis by activating the peroxisome proliferator-activated receptor gamma coactivator 1α (PGC-1α) pathway (Hsu et al., 2022). It also activates protective mitochondrial autophagy via pathways such as AMPK to initiate “clearance” of mildly damaged mitochondria, thereby enhancing mitochondrial quality control during the injury phase. Concurrently, it regulates proteins such as Drp1 and Mfn2, inhibiting excessive mitochondrial fission during reperfusion while promoting fusion to maintain mitochondrial network stability (Hentia et al., 2018). The mechanism by which hyperbaric oxygen pretreatment protects mitochondria can provide valuable insights into the protective mechanism of ozone pretreatment on mitochondria. On the other hand, rational ozone use may be readily achievable in clinical settings, suggesting its potential as an alternative to “supplementary” therapy for I/R injury. While reasonable ozone use is generally considered non-toxic to humans, there are reports of adverse reactions to medical ozone treatment. Avcı et al. reported the case of a female patient with Meniere’s disease who developed sudden bilateral cortical blindness after inhaling ozone (Avcı et al., 2015). Another female patient with lumbar disc herniation experienced sudden frontal headaches and bilateral blindness during an ozone disc injection procedure (Corea et al., 2004). The adverse reactions reported in a study of COVID-19 adjunctive therapy included injection site pain, elevated liver enzymes (AST/ALT), headache, and hyponatremia (Setyo Budi et al., 2022). These adverse events likely stem from improper concentration, dosage, or administration methods and warrant further investigation. First, the lack of high-quality case reports and insufficient large randomized controlled trials (RCTs) in this field, in addition, the scarcity of direct comparative studies on risks across different administration routes, the absence of adverse event database information, and the lack of more detailed and authoritative clinical practice guidelines all provide recommendations for future clinical practice and research directions in this field.

Most studies (listed in the table) confirm that ozone treatment before ischemia or reperfusion can mitigate organ damage, thus offering an effective intervention for managing I/R injury following sudden ischemic events (cerebral infarction, myocardial infarction, testicular torsion, etc.) and unavoidable blood flow interruption during surgery (orthopedic and extremity surgeries, liver resection, organ transplantation, etc.). Appropriate ozone use not only prevents I/R injury but also protects distal organs from ischemic damage, promotes tissue/organ regeneration and recovery, and improves organ survival post-transplant (Koçyiğit et al., 2018). For instance, medical ozone treatment can reduce immune rejection after heart transplantation and improve the survival rate of the transplant (Stadlbauer et al., 2008). In another study, ozone was found to alleviate arrhythmias and improve myocardial function, highlighting its multifunctionality as a therapeutic agent (Di Filippo et al., 2010a). Furthermore, ozone pre-conditioning provides long-term protection against renal I/R injury and reduces the formation of renal fibrosis (Wang et al., 2014). Despite these promising outcomes, the current research on medical ozone treatment has several limitations. First, the mechanisms of medical ozone treatment have been largely elucidated using biochemical markers and histological methods, rather than genomic or proteomic analyses. Second, most studies have focused on the role of established antioxidant pathways (e.g., the Nrf2 pathway) and have neglected the potential impact of other pathways (e.g., the MAPK pathway) on ozone effects. Third, the research on medical ozone treatment is predominantly based on rodent models, and there is a dearth of primate or phase I clinical trials to evaluate efficacy and potential risks. Fourth, medical ozone treatment in animal models usually involves intraperitoneal or intestinal perfusion, and the limited exploration of alternative delivery methods hinders comparisons to determine optimal administration protocols. Finally, there are limited studies on the optimal therapeutic concentrations of ozone, timing of administration (pre-treatment vs. reperfusion phase), and organ-specific variations. Furthermore, the studies of protection against ischemia-reperfusion injury by ozone are focused primarily on the heart, kidneys, and liver. The studies on other complex ischemia-reperfusion injuries encountered in clinical practice remain relatively scarce, which also provides researchers with broader avenues for exploration.

The studies outlined in this review provide a robust theoretical foundation for the clinical application of ozone against I/R injury. Although some studies have yielded unsatisfactory outcomes, this may be attributed to variations in the administration methods, timing, concentration, and dosage of ozone, thus necessitating further research. While established clinical therapies such as hyperbaric oxygen therapy and ischemic pre-conditioning are effective against I/R injury, ozone pretreatment exhibits comparable or even superior protective effects. In addition, ozone has also shown satisfactory results when compared to potential therapeutic drugs. Altogether, these findings support the rational clinical use of ozone for I/R injury and related conditions, as well as further exploration of its therapeutic potential.
